# Cancer Classification at the Crossroads

**DOI:** 10.3390/cancers12040980

**Published:** 2020-04-15

**Authors:** Antonino Carbone

**Affiliations:** Centro di Riferimento Oncologico di Aviano (CRO), IRCCS, Via F. Gallini 2, I-33081 Aviano, Italy; acarbone@cro.it; Tel.: +39-0434-659-085; Fax: +39-0434-659-370

**Keywords:** cancer, classifications, cancer diagnosis, cancer genomics, cancer management

## Abstract

Internationally accepted classifications of malignant tumors, developed by the World Health Organization (WHO) and the Union for International Cancer Control (UICC), are based on the histotype, site of origin, morphologic grade, and spread of cancer throughout the body. The WHO classifications are the foundation of cancer diagnosis and the starting point for cancer management. Starting in 2000, the WHO classifications began to include biologic and molecular–genetic features. These developments are having a strong impact on cancer diagnosis and treatment, and this impact is amplifying, given the advances in cancer genomics. Molecular–genetic profiling can be used to refine existing classifications of tumors and, for a small but increasing number of cancers, even determine the treatment irrespective of histotype. Here I discuss how cancer classifications may change in the era of cancer genomics.

## 1. Introduction

The global incidence of cancer has increased to an estimated 18.1 million new cancer cases in 2018 [1] and is expected to reach 25 million by 2040 [2] with the largest impact in low- and middle-income countries [2]. Cancer care is distributed unevenly among countries [3], and, due to its complexity, only specialized hospitals can enact state-of-the-art clinical management.

Currently, the diagnosis of a tumor and its management is based on the confirmation of its malignancy, and on its site of origin, histotype, grade, and spread throughout the body. These features are defined in the classifications of malignant tumors developed by the World Health Organization (WHO) and the Union for International Cancer Control (UICC). These classifications, created to help management decisions, represent a consensus among pathologists, radiologists, and clinical oncologists, and are based on epidemiological, morphological, phenotypic, and biological data. Application of these classifications requires instrumentation and skills for clinical pathology, molecular testing, and diagnostic imaging, and so depends on the socioeconomic context in which patients and their clinicians find themselves [4]. It is, therefore, evident that, in clinical cancer management, tumor classifications cannot be uniformly applied across the world today. However, in the era of precision medicine and cancer genomics [5], the application of these internationally accepted classifications is considered obligatory as the starting point for cancer management. Clinical guidelines rating strength of evidence indicate which gene tests are recommended in defined clinical settings, and how management decisions are impacted by gene test results. Here I describe existing cancer classifications and discuss how they may change in the future, given the advances in cancer genomics.

## 2. Established Tumor Classifications and Grading Systems

Tumors are traditionally classified four ways: (I) broadly, by tissue, organ, and system; then by (II) specific type, and (III) grade according to WHO classifications; and (IV) finally by spread according to the Tumor Node Metastasis (TNM) system. These classifications have had a major impact on clinical oncology, cancer research, and the training of oncologists and pathologists. The classifications help residents in pathology learn the clinical importance of analyzing surgical specimens, assessing the radicality of tumor excision, and determining the extra-organ tumoral spread by examining the tumor margins, resection margins, and lymph nodes. Moreover, they help pathologists across the world learn the scientific terminology and the established diagnostic protocols.

In the broad tumor classifications organized by tissue or organ of origin, hematological cancers are distinguished from solid neoplasms, which are further classified as carcinomas, if they originate from epithelial cells of the skin, gastrointestinal tract, internal organs, and other anatomical sites; sarcomas, if they derive from muscle, adipose, bone or blood vessels; lymphomas, if they originate in the lymphoid tissue; and so on. Beyond these main types, numerous tumor histotypes have been defined and codified, since 1957, in the WHO Classification of Tumours, a series of volumes each dedicated to a different organ system (15 volumes are expected in the fifth edition of 2019–2020). These reference volumes for the histopathological classification of tumors also present concise information on each tumor’s genetics, epidemiology, clinical and imaging findings, risk factors, and prognosis, as a guide for the diagnosis.

Tumors are then graded as a component of prognosis [6]. Tumor grading, according to the WHO system, combines cytological features (e.g., extent of cellular differentiation and presence of dysplasia) and morphological–structural observations (e.g., mitotic count and necrosis). The grade is expressed numerically, generally from a low grade of 1, indicating a high level of cellular differentiation, to a high grade of 3 (poorly differentiated or undifferentiated). Some tumors have their own grading systems. For example, the grading systems for prostate carcinoma and melanoma give particular importance to tumor architecture.

Finally, tumors are classified according to their stage, i.e., the extent of spread throughout the organism. The most widely used system for scoring tumor spread is the TNM Classification of Malignant Tumors [7]. First developed in 1958 by the UICC, the TNM system is now in its eighth edition. It rates the size or extent of the primary tumor (T), the degree of spreading to lymph nodes (N), and the presence of distant metastases (M); each of these three categories has several numbered classes. The TNM system facilitates the exchange of information about tumor development among cancer hospitals, helps in the planning of treatment, and the assessment of outcomes, and is useful in screening programs.

Whereas these standard tumor classifications focus on tissue or organ of origin and histopathological, clinical, and epidemiological data, the WHO classifications began to include molecular–genetic features of tumors, starting from the third edition in 2000. The extent of inclusion of molecular pathology features, however, is limited because, internationally, few laboratories had such diagnostic abilities. Nonetheless, these developments are having a profound impact on how cancer is diagnosed and treated.

## 3. Impact of Molecular–Genetic Data on Tumor Classification and Treatment

A tumor is the final stage of a multistep genetic process that involves “cancer genes” [8] and the inhibitory and stimulatory signals that they produce [9]. In hematopoietic and lymphoid tissue, the definition of neoplasia relies on molecular tools to distinguish monoclonal from polyclonal cell proliferation. In most tumors, biological markers are now being used to identify specific tumor types according to the presence or absence of certain genetic lesions and to refine the classification of some tumors. For example, some breast tumors express high levels of receptors for the hormones estrogen and progesterone and of HER2 (a receptor tyrosine kinase of the ErbB family), while other breast tumors are negative for these markers [10]. These biological differences are reflected in differences in clinical behavior, allowing tumor subtypes to be defined.

While molecular profiling can refine existing classifications of tumors, it can also call into question those classifications because many tumors of different tissue origins share genetic alterations. For example, carcinomas of different tissue origins share genomic and transcriptomic derangements depending on whether they derive from the luminal or basal stratum [11]. The MYC gene, which encodes a transcription factor, is translocated in lymphomas and amplified in some carcinomas of the breast, ovary, stomach, lung, and skin [12]. MYC amplification is associated with aggressive disease, resistance to treatment, and poor outcomes [13]. Aberrations in this and other genes cannot be used to define tumor subtypes, but they can help predict outcome and guide treatment.

Pathological classification and molecular–genetic profiling can be used together in choosing the treatment strategy. Let us take breast cancer as an example on how molecular data and pathological classifications can be integrated for tumor diagnosis and prognosis. The standard classification identifies the morphologic grade and clinicopathological stage of the tumor, while the molecular–genetic characterization provides information on estrogen receptor expression (by immunohistochemistry), *HER2* (*ERBB2*) gene amplification status (in situ hybridization), *HER2* activating mutations or *PI3K*CA mutations (DNA sequencing), proliferative index (MIB1 immunohistochemistry), and RNA expression signatures with prognostic value (i.e., prognostic gene signatures). The treatment can include an estrogen antagonist (when oestrogen receptors are overexpressed), a monoclonal antibody that blocks HER2 activity (when *HER2* gene is amplified), or chemotherapy (when the grade, TNM stage, or gene expression profile indicates a high risk of relapse [14]). Another example with expected clinical application is the case of peripheral T cell lymphomas not otherwise specified: this heterogeneous group of lymphomas has recently been subclassified, on the basis of gene and protein expression profiles, into two subtypes with distinct prognoses [15]. Thus, molecular information is helping to distinguish tumors into subtypes for which different treatments can be developed.

Noteworthy, there are clinical examples of the same genomic alteration displaying different theranostic associations, dependent on the tissue/tumor type, such as BRAF V600E mutations in melanoma compared to colorectal cancer. For a small but increasing number of locally advanced or metastatic cancers, the molecular–genetic findings determine the treatment, irrespective of the morphological–pathological findings. For example, more than 20 different tumors have a chromosomal rearrangement fusing a neurotrophic tropomyosin receptor kinase (NTRK) gene with another gene, increasing kinase activity; these tumors can now be treated with drugs targeting NTRK-fusion kinases [16]. Recently, gene fusions involving NRG1, which encodes the growth factor neuregulin-1, have been found in 11 different tumor types [17]. Because these fusions have an activating effect on neuregulin-1, which itself activates ErbB receptor tyrosine kinases, tumors whose driving mutation is an NRG1 fusion should be treatable with ErbB tyrosine kinase inhibitors. Ongoing basket trials [18,19], which test one molecularly targeted treatment against different tumors sharing a particular molecular defect, will say whether such lineage-independent (“tissue agnostic”) therapy will be the future for oncology [20].

**Figure 1:**
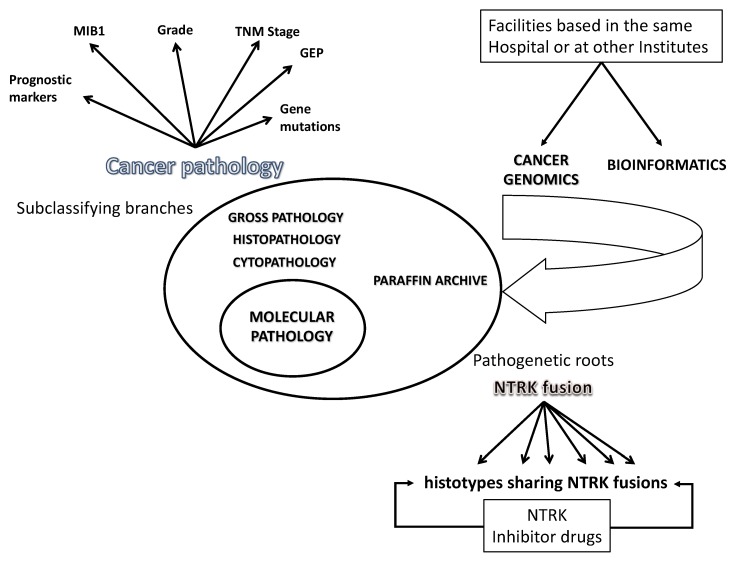
Standard classification and genomic profiling in a contemporary department of pathology. Facilities for cancer diagnosis and research carry out conventional histopathological analyses as well as biological and molecular–genetic analyses. The core structure also receives data from genomic and bioinformatics research facilities, either based in the same hospital or at other institutes. Standard pathology classification for cancer includes morphology, immunohistochemistry, and pTNM stage. Molecular profiling can refine this classification. Different tumor histotypes may share a genetic mutation, making them susceptible to treatment with the same drug. The figure illustrates how some tumors of various histotypes, grades and stages may be driven by a chromosomal rearrangement fusing a neurotrophic tropomyosin receptor kinase (NTRK) gene with another gene. Histotypes sharing NTRK fusions include thyroid carcinoma, melanoma, gastrointestinal stromal tumor, lung carcinoma, colon carcinoma, salivary gland tumor, central nervous system tumors, soft tissues sarcoma, infantile fibrosarcoma, and others (not shown). Abbreviations. GEP, gene expression profile; pTNM, pathologic TNM.

For pathologists, these different approaches to classifying tumors for treatment decisions already have a profound professional impact. Pathology laboratories in cancer centers are faced with the choice of dividing into distinct departments for conventional diagnostics and cancer genomics, or transforming into a modern diagnostic service with a core facility for pathological, biological, and molecular–genetic analyses and relying on other laboratories for more specialized services and research support (Figure 1). Next generation sequencing (NGS) studies had deciphered the genetic mutation landscape in cancer and recognized driver genes associated with distinct histotypes (reviewed in [21]). Gene-panels have been developed to screen these genes in cancer patients for diagnosis, prognosis, and therapeutic implications. Accurate information is possible using small pre-surgical biopsies (reviewed in [21]). In this regard, it should be highlighted that health disparities, such as higher death rates in people from low socioeconomic groups, still remain. These disparities are substantially caused by diagnostic delay and are related to the global variation in the availability and/or accessibility of diagnostic tests for cancer.

## 4. Conclusions

We have arrived at a crossroads in the classification of malignant tumors. Our classification systems have always evolved and will continue to evolve as new scientific data emerge. We must decide if it is appropriate to continue to use the familiar WHO and TNM systems [22] or if it is opportune to define new tumor classes according to the mounting molecular–genetic data, looking to the future.

If the second route is chosen, genomic data will be used to determine the treatment irrespective of histotype. The feasibility of this approach will be revealed by the outcomes of ongoing basket trials. The new cancer classifications that result should be accompanied by databases of the most frequent genomic alterations in common tumors, together with clinical information and data on the response to treatments. A clinical resource (e.g., ClinVar database) of this sort could provide oncologists with constantly updated information to help manage cancer throughout the world.

Taking this route is a useful, and probably necessary, decision considering the potential of new drugs targeted to specific genetic defects. The main risk associated with this decision, from the viewpoint of clinical management, is that of moving fast on a road lacking warning signs for obstacles and hazards. Nonetheless, given that the era of cancer genomics is certain to persist, it is wise not to resist change but to accept the new diagnostic test (i.e., in premalignant neoplasia [HPV DNA], in distinguishing myeloproliferative neoplasias or myelodysplasia from reactive myeloid proliferations) and use the new therapeutic approaches with caution, applying them for the moment to cases of locally advanced or metastatic tumors, all the while learning to drive along this new highway.
